# Lactate-Mediated Signaling in the Brain—An Update

**DOI:** 10.3390/brainsci13010049

**Published:** 2022-12-27

**Authors:** Barbara Vaccari-Cardoso, Maria Antipina, Anja G. Teschemacher, Sergey Kasparov

**Affiliations:** 1 School of Physiology, Pharmacology and Neuroscience, University of Bristol, Bristol BS8 1TD, UK; 2 MEDBIO, Immanuel Kant Baltic Federal University, Universitetskaya Str., 2, 236041 Kaliningrad, Russia

**Keywords:** lactate, astrocyte, signaling, metabolism, receptor

## Abstract

Lactate is a universal metabolite produced and released by all cells in the body. Traditionally it was viewed as energy currency that is generated from pyruvate at the end of the glycolytic pathway and sent into the extracellular space for other cells to take up and consume. In the brain, such a mechanism was postulated to operate between astrocytes and neurons many years ago. Later, the discovery of lactate receptors opened yet another chapter in the quest to understand lactate actions. Other ideas, such as modulation of NMDA receptors were also proposed. Up to this day, we still do not have a consensus view on the relevance of any of these mechanisms to brain functions or their contribution to human or animal physiology. While the field develops new ideas, in this brief review we analyze some recently published studies in order to focus on some unresolved controversies and highlight the limitations that need to be addressed in future work. Clearly, only by using similar and overlapping methods, cross-referencing experiments, and perhaps collaborative efforts, we can finally understand what the role of lactate in the brain is and why this ubiquitous molecule is so important.

## 1. Introduction

Our appreciation of the diversity of the actions and interactions of L-lactate (Lac) in the brain continues to expand, and with it, our understanding of the potential mechanisms by which Lac fulfils its diverse roles. While several recent reviews have discussed specific aspects of central Lac effects [1,2,3,4], the field is still full of controversies. Here, we present only a handful of studies put forward by different groups, in order to highlight potential new avenues of viewing previous findings and mechanistic interpretations. The selectivity of tools to study Lac actions is gradually improving but their limitations should be considered when interpreting effects on pathways with complex interconnectivity such as those in which Lac is involved.

**Figure 1:**
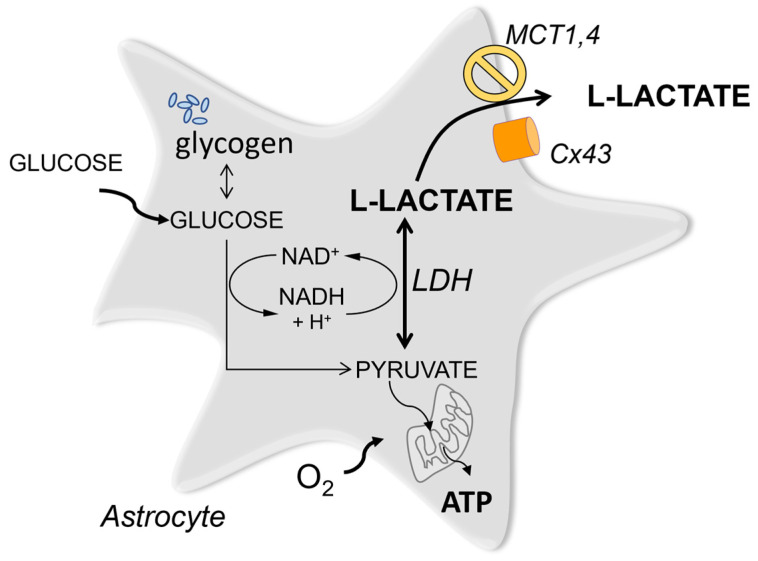
Interconversion of lactic and pyruvic acid is mediated by LDH and recovers NAD^+^ used in glycolysis. Glucose for glycolysis is imported from the periphery or/and recruited from glycogen under conditions that stimulate glycogenolysis.

Following glycolysis, Lac is generated from pyruvate by lactate dehydrogenase (LDH), an enzyme that operates in both directions, using NADH to reduce pyruvate into Lac (Figure 1). Even though LDH isoforms differ in their interconversion rates, both LDHA and LDHB are thought to establish an equivalent pyruvate-Lac equilibrium under steady-state conditions [5]. As such, Lac cannot be metabolized any further. It is either released to the extracellular space and shared with other cells, or is reconverted by LDH to pyruvate which then can enter the TCA cycle in the mitochondria.

All Lac release mechanisms identified so far are gradient-dependent. These involve monocarboxylate transporters (MCT) of which isoforms 1 and 4 are predominantly expressed by astrocytes and MCT2 is mainly found in neurons [6]. In addition, Lac release from astrocytes through connexin hemichannels has been implied by a number of publications [7,8]. A Lac-permeable ion channel which could be activated by depolarization and positively modulated by Lac has also been described [9] and further routes may potentially exist. In the brain, Lac is released by both neurons and astrocytes but it is well established that astrocytes produce and release more Lac than neurons [10]. This creates a gradient of Lac from astrocytes towards the extracellular space and neurons [11,12]. As can be seen from the following, some of the proposed mechanisms require Lac entry into neurons in the vicinity, others postulate that Lac acts on receptors located on the cell surface. Our aim here is to highlight the remaining questions, discuss the usefulness of commonly used tools, and suggest interesting avenues to explore further.

## 2. Mechanisms Which Are Attributed to Lac Entry into Target Neurons

The concept of Lac being passed on between different cell types is well established for peripheral tissues [13]. For the brain, the hypothesis of an analogous Lac shuttle operating between astrocytes and neurons was proposed decades ago [14], originally, as a mechanism to subsidize neurons with energy under conditions of high metabolic demand such as periods of active firing of action potentials. The supporting evidence for the Lac shuttle hypothesis, including why Lac may be a preferred substrate to glucose, can be found in publications from P.J. Magistretti and colleagues [1,4]. Up to this day, this hypothesis remains a matter of debate with strong arguments for [12] as well as against it [15,16,17,18]. Perhaps one of the more contentious aspects of the shuttle hypothesis is the question of why astrocytic Lac, once transferred into neurons, should be used in preference to glucose for ATP generation. Tracing experiments with ^13^C-labelled glucose and other compounds have indicated that in the brain, in contrast to peripheral tissues, glucose rather than Lac is the primary source of TCA metabolites [19]. Other studies also argued that glucose is, in fact, the preferred neuronal source of energy, at least under physiological conditions and normal glucose concentrations [15,16,20]. Hence, the relative importance of astrocytic Lac as a source of neuronal ATP is still controversial.

### 2.1. Mechanisms Primarily Linked to Increased ATP Production in Neurons

The hypothesis that astrocytic Lac is the preferred substrate for ATP generation in neurons goes back to the initial Lac shuttle concept as mentioned above [14,21,22]. In some studies, this mechanism is implied, rather than demonstrated directly. Typically, conclusions are based on the sensitivity of the recorded effects to LDH blockade—as, without conversion into pyruvate, Lac cannot be used for ATP production—or to inhibition of Lac transport through MCT, for example by 4-CIN or, in some cases, antisense oligonucleotides (AS-ODN) targeted at the expression of MCT. Often in such studies, it is not possible to confidently exclude contributions of NAD^+^/NADH ratio changes (see next section) or of intracellular acidification.

Studies from two laboratories as examples of Lac-dependent processes that are attributed to Lac’s caloric value for neurons are discussed in this section. In 2011, the group of C. Alberini published a paper implicating Lac shuttle in memory at the level of the hippocampus [23]. More recently, this group extended their arguments and reported that astrocyte-derived Lac may affect excitatory and inhibitory neurons in the hippocampus via modulation of mRNA translation [24]. They showed that microinjections of 1,4-dideoxy-1,4-imino-D-arabinitol (DAB) into rat hippocampus disrupted memory, which was rescued by co-injection of pyruvate but not by an equicaloric quantity of glucose [24]. Of note, sensitivity to DAB, an inhibitor of glycogen phosphorylase and synthase, can point to the involvement of astrocytic Lac production in a process. However, it does not distinguish between the actual Lac targets mediating the effect. Rescue by pyruvate implies import via MCT and a preference for monocarboxylates over glucose. Interestingly, while pyruvate can be transported by the same MCT as Lac, and has an equivalent caloric value, it will have the opposite effect on the NAD^+^/NADH ratio (see section below) since LDH will use NADH to re-establish the Lac-pyruvate equilibrium.

In order to selectively inhibit Lac export from astrocytes, AS-ODN was used to suppress the expression of MCT1 and MCT4 [24]. AS-ODN interfere with the translation of specific mRNAs and thus the synthesis of the respective proteins. Long-term memory was disrupted within only one hour of AS-ODN injection. This effect was also rescued by pyruvate, which can be explained by the fact that neurons express mainly a different type of MCT, MCT2 [24]. Given that the half-life of most proteins in mammalian cells, including in neurons and glia, by far exceeds one hour, the reduction of MCT protein levels here is a surprising observation [23,24,25] and may potentially point to off-target effects of this treatment which merit further study. The authors also assessed the impact of the Lac shuttle on mRNA translation, an important step in long-term memory and, as they argue, requires a significant amount of ATP. They found that the training protocol significantly increased the incorporation of puromycin into newly made proteins (so-called SUnSET protocol) as tested 2 h after training. This increase was blocked by DAB and, therefore, is dependent on glycogenolysis in astrocytes. Moreover, both Lac and pyruvate were able to rescue increased translation after DAB administration. Additionally, expression of the immediate early gene Arc/Arg3.1 was also dependent on Lac supply. The take-home message from this work is that Lac derived from glycolysis in astrocytes is important to energetically support the translation of proteins required for memory formation.

Another study that argues for the importance of the caloric value of astrocyte-derived Lac looked at nociceptive transmission at the level of the dorsal horn of the spinal cord in mice [26]. The authors used a chemogenetic approach to activate astrocytes in the dorsal horn whereby they employed a Gq coupled Designer Receptors Exclusively Activated by Designer Drugs (DREADD). DREADD was expressed unilaterally using an adeno-associated virus with a GFAP promoter. After activation of DREADD by clozapine-N-oxide (CNO) administration, the extracellular concentration of Lac was found raised from ~1.2 mM to ~2.0 mM. Mechanosensitivity of the hind paw greatly increased after CNO injections and stayed high for several hours. This effect only occurred on the side which expressed DREADD. The authors used 4-CIN to block MCTs and showed that it prevented sensitization caused by CNO. Conversely, intrathecal injections of Lac caused pain sensitization and induction of several immediate early genes, markers of neuronal activation. These effects, too, could be blocked by 4-CIN, implying the need for the transfer of Lac into neurons. Finally, the study looked at mechanical allodynia caused by partial nerve ligation. Several treatments targeting astrocytic Lac production had profound anti-nociceptive effects, including LDH inhibition by isosafrole, inhibition of astrocytic TCA cycle by fluorocitrate (FC) and MCT blockade by 4-CIN, mentioned above. In some cases, the threshold of mechanosensitivity almost returned to the level of the contralateral control paw. While the paper is consistent with a Lac shuttle mechanism between astrocytes and neurons in nociception, it does not provide direct evidence that it is the caloric value of Lac which is important in these processes, and not, for example, a shift in NAD^+^/NADH ratio or pH. A further complication is that 4-CIN, while an effective inhibitor of cellular Lac ex- and import, may also effectively deprive neurons (and astrocytes) of ATP generated by the mitochondria (see Section 2.3 below). Finally, the effects of FC are somewhat difficult to interpret as, in addition to affecting the mitochondrial TCA cycle, it depolarizes astrocytes [27], thereby affecting a host of potential-dependent transporters (see further comments below).

Overall, the body of studies which support the use of Lac for energy generation in preference to glucose is substantial, for example [21,28], but is it always preferred to glucose and why? A recently published study from the L. Venance group offers an essential clue which may explain the existing controversies [29]. Here, experiments in vitro, in vivo and mathematical modelling are combined to carefully dissect which conditions favor the utilization of glucose vs. Lac. The authors used two types of protocols, for example, in vitro, a high frequency (100 Hz) 5× theta burst stimulation that should require more energy for the generation of LTP and, for comparison, spike timing–dependent plasticity (STDP) where the frequency of stimulation is relatively low. Both forms of plasticity are dependent on NMDA receptors. It was shown that while the high frequency LTP requires Lac provision, STDP does not. In vivo, the authors use a simpler novel object recognition task where the rat needs to detect one new object and compare it with a more complex test (object in place) where several objects have been moved in the arena. Here, the simple test is not sensitive to oxamate while the more challenging test is, again pointing to the preferential use of Lac in situations of high energy demand. These experiments are matched by mathematical modelling. This study demonstrates that, while Lac (provided largely by astrocytes) is required to support synaptic activity when the energy consumption is high, the conditions of the experiment are paramount.

### 2.2. Mechanisms Attributed to NAD^+^/NADH Ratio Changes

Apart from serving as a potential energy substrate, the import of Lac by MCT has several other effects on receiving cells. First of all, LDH converts excess Lac into pyruvate (Figure 1), increasing NADH and decreasing the NAD^+^/NADH ratio as a result.

#### 2.2.1. Potentiation of NMDA Receptor Activity in Memory Formation or Retention

The hypothesis that a change in NAD^+^/NADH ratio can affect NMDA receptors originates from the laboratory of P.J. Magistretti [30,31]. Initially, it was demonstrated that high concentrations of Lac (2.5 mM–20 mM) can trigger the expression of several immediate early genes and potentiate NMDA currents in cultured neurons. As mentioned above, the entry of Lac into the cells raises NADH levels. The study inferred that this increase in NADH is ultimately responsible for the potentiation of NMDA currents and increased expression of immediate early genes. Both events may be expected to affect memory formation. The actual mechanism of how the NAD^+^/NADH ratio changes the function of NMDA receptors was not unequivocally identified in this paper. Bearing in mind that a strong potentiation of NMDA currents could potentially lead to excitotoxicity, later work by the same group suggested that Lac may counteract any neurotoxic effects by neuroprotective actions by supplying neurons with energy when converted into pyruvate [32]. The balance of the two actions was suggested to depend on the strength of the glutamatergic stimulation.

**Figure 2:**
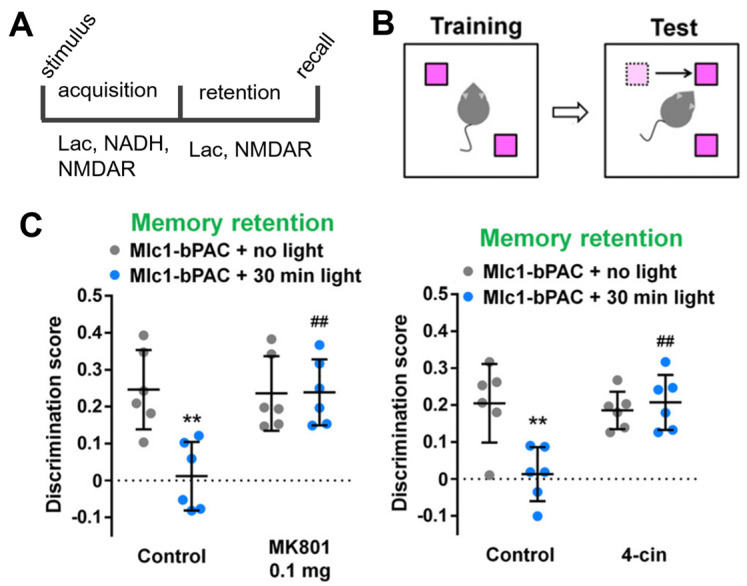
(**A**) Astrocyte-derived Lac is suggested to be involved in memory formation at multiple stages. (**B**) Mice were trained on a memory paradigm in which they spent more time around an object that had been moved from its previous location. Astrocytes were stimulated either during training or 24 h later using bPAC. Discrimination was assessed on day 3. Modified from [34]. (**C**) Memory test on day 3 demonstrated that optogenetic stimulation of cAMP on day 2 blocked the ability of mice to discriminate object location (second sets of data points). However, both MK 801 and 4-CIN prevented the effect of bPAC activation (fourth sets of data points), and mice performed similarly to animals which were not light stimulated (first sets of data points). Without light stimulation, MK801 and 4-CIN did not change the discrimination scores on day 3 (third sets of data points). ** *p* < 0.01 vs. the Mlc1-bPAC + no light + vehicle group; ## *p* < 0.01 vs. the Mlc1-bPAC + light + vehicle group by Tukey’s test after one-way ANOVA. Modified from [34].

The concept that NMDA receptors mediate the effects of Lac was later developed in a study that used bPAC, a light-sensitive adenylate cyclase from *Beggiatoa*, to optogenetically stimulate cAMP production in astrocytes [33,34]. In many cells, including astrocytes, cAMP elevation is a trigger for energy mobilization and glycogenolysis. Since astrocytes are essentially the only glycogen-containing cells in the brain, they can mobilize this store upon PKA activation by cAMP and rapidly deliver Lac into the extracellular space [35,36]. Although the link to the NAD^+^/NADH ratio was not specifically proven, this study reported that raising cAMP in astrocytes disrupted mouse behavior in the object location memory paradigm (Figure 2). Interestingly, cAMP increased memory scores when the light was applied during training (consistent with the general concept of Lac acting on canonical NMDA-dependent mechanisms of memory formation). However, the same protocol inhibited memory if light stimulation was performed on day two after training and before memory was tested on day three (Figure 2). The authors discuss these effects as Lac having a positive impact on memory formation and a negative impact on memory retention.

The arguments made in favor of the role of Lac shuttle were that the negative effect of cAMP on memory retention 24 h after training could be antagonized by 4-CIN, as well as by an NMDA receptor blocker (MK 801; Figure 2).

Altogether, while the effects of these drugs allow a clear-cut interpretation and the authors focus their discussion on the Lac shuttle hypothesis [34], several questions remain open. It seems that when cAMP is raised in astrocytes, it can have opposite effects on memory acquisition and storage, but in both cases, Lac shuttle and NMDA receptors are involved. Unfortunately, the effects of NMDA or MCT blockade on memory acquisition during or immediately after training, which may be expected to be the most critically NMDA receptor-dependent steps in memory formation, were not demonstrated in this paper. Additionally, the mechanism of memory disruption when astrocytes were activated 24 h after training is not immediately obvious—how should NMDA activation or Lac shuttling inhibit memory storage at that stage?

Another study confirmed that activation of astrocytes, in this case using a G_q_-coupled DREADD or a G_q_-coupled opsin (OptoG_q_), increased memory when performed during acquisition [37] but these stimuli were ineffective when applied during the test for memory recall. This study well illustrates one of the great challenges for elucidating the role of astrocytes in memory formation which is to understand the specific localization of Lac action with respect to the “memory trace”: How can an activated astrocyte or a group of astrocytes modulate a specific memory trace, given that each astrocyte contacts numerous neurons, potentially many thousands of synapses? Moreover, Lac almost certainly can spread between astrocytes via gap junctions, which would further diffuse the signal within the network. Can astrocytic modulation be targeted to individual synapses which are contacted by distinct end feet of the same astrocyte? Or is the role of astrocytes to provide a wide-scale change in the extracellular concentration of metabolites such as Lac, ATP, glutamate, etc. that results in more general network modulation? Under which physiological conditions could one expect the activation of large pools of astrocytes in the hippocampus and what would be the mechanism? If, as per [34], such activation occurs, would that elicit amnesia covering the preceding 24 h? These are exciting questions and, clearly, a lot of work still needs to be done to explain how the stimulation of astrocytes affects memory formation and retention and whether there may be specific mechanisms for compartmentalization of intra- and inter-cellular Lac signaling in astrocytes.

#### 2.2.2. Potentiation of NMDA Receptor Activity in Memory Formation or Retention

An interesting mechanism related to the NAD^+^/NADH ratio was found in carotid body glomus cells [38]. Although not central, these cells have all the classical features of neurons and deserve mentioning here. The paper proposes another hypothesis which also requires Lac import and conversion by LDH. It demonstrated that the change in cytosolic NAD^+^/NADH ratio may cause activation of non-selective cation channels that are sensitive to the non-selective cation channel blocker 2-APB and belong to the transient receptor potential (TRP) channel family. As a result, neurons depolarize and start firing action potentials, leading to Ca^2+^ entry and elevation of intracellular Ca^2+^ concentration. Entry of Lac is mediated by a co-transport with protons and therefore decreases intracellular pH. The authors argue that acidification increases mitochondrial reactive oxygen species production, which further activates glomus cells [38]. This is a rare example of a study where Lac-induced acidification was actually taken into account.

### 2.3. Methodological Considerations of Commonly Employed Tools in the Study of Lac Transfer from Astrocytes into Neurons

In order to prove the involvement of the Lac shuttle, any effect of Lac needs to be primarily sensitive to the blockade of Lac entry into neurons. Inhibition of Lac-pyruvate interconversion by LDH enzymes is also used as an argument to support the notion of Lac shuttle. However, block of the transporters by 4-CIN is bound to inhibit Lac release from astrocytes, while block of LDH should also block the generation of Lac in astrocytes. Drugs which are currently used are not selective to a specific location of any of these processes.

The MCT blocker 4-CIN (α-cyano-4hydroxycinnamate or CHC) is used in many studies on this topic and will block astrocytic release as well as neuronal uptake of Lac. However, it was established long ago that 4-CIN is two orders of magnitude more potent at inhibiting pyruvate transport into mitochondria than at inhibiting Lac transport across the plasma membrane (reviewed in [39]). Therefore, while 4-CIN may well be an effective inhibitor of Lac import into the cells, blockade of pyruvate uptake by the mitochondria could shift the LDH reaction towards Lac formation and lead to increased levels of intracellular Lac. Moreover, 4-CIN could also prevent astrocytes and neurons from using the resultant pyruvate for oxidative phosphorylation in the mitochondria, thus additionally depriving cells of energy. In addition, it has been shown that 4-CIN application, probably by inhibiting the mitochondrial pyruvate transporter, can cause profound acidification in neurons, which is an additional complication [40]. Newer, and potentially more selective, AR-C compounds are available for the inhibition of MCT [41]. Knock-down of astrocytic or neuronal MCT expression using AS-ODN is a potentially more selective approach which has been used in some studies [24]. However, the reported rapidity of the AS-ODN action, which lowered the expression of MCT proteins within one hour is rather surprising. The half-life of MCT proteins must be taken into account to further validate this approach. The effects of FC are very difficult to predict and interpret. Fluoroacetate, which is converted into FC in the cells, is listed as a highly toxic compound and is even considered among chemical warfare agents [42]. FC application results in the accumulation of citrate on one hand and a reduction of glutamine, the precursor of GABA, on the other [43,44]. Obstruction of the TCA cycle leads to a rapid increase in lactate and pyruvate extracellular levels [42]. At the same time, because of the failure of the ATP-driven Na^+^/K^+^ exchanger, astrocytes depolarize and this can cause the release of various signaling molecules, such as purines or possibly glutamate, which would result in the activation of adjacent neurons [27]. It follows that, because of its multiple effects, the interpretation of FC experiments can be problematic.

Finally, as mentioned earlier, we should not disregard the fact that entry of Lac into the cells through MCT must necessarily lead to acidification of the cytoplasm by the co-imported protons. These effects may become particularly prominent when concentrations of Lac are used that exceed the physiological range, which is thought to be below 2 mM (reviewed in [45]). Indeed, in some of the studies mentioned above, a 40 mM solution of Lac was microinjected locally into the tissue, or concentrations of 10–20 mM Lac were applied to the cells [26,31]. Numerous studies where Lac was expected to enter neurons disregarded changes in pH, and we think this might be more important than is currently appreciated.

## 3. Cell Surface Receptor-Mediated Signaling by Lac in the Brain

**Figure 3:**
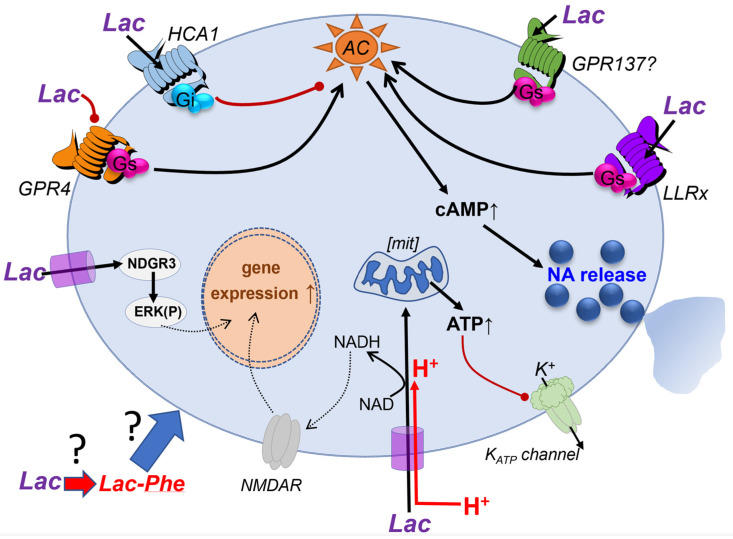
Summary of putative Lac receptor-mediated signaling mechanisms in brain cells. Lac transported into the cell can be metabolised and/or influence gene expression, e.g., via NMDA receptor modulation or ERK pathway activation. Increased ATP levels may inhibit KATP channel activity and decrease cell excitability. Lac may also act via surface GPCR to stimulate or inhibit neurones. The effects of acidification caused by protons co-imported via MCT require clarification. We also hypothesise that Lac can be converted into Lac-Phe in the brain, the implications of which have yet to be discovered. Black arrows—stimulatory action; red lines—inhibitory. AC—adenylate cyclase; NA—noradrenaline; NDRG3, ERK(P)—phosphorylation of extracellular signal-regulated kinases; mit—mitochondria. Modified from [48].

It is well established that Lac can affect cell function via cell surface G-protein coupled receptors (GPCR) without the need to access the cytoplasm (Figure 3). Lac has its cognate receptor, registered by IUPHAR (https://www.guidetopharmacology.org/ (accessed on 1 December 2022)) as HCA1 (previously known as Lactate receptor 1, LACR1, GPR81, GPR104). It is encoded by the gene HCAR1. The physiological role of HCA1 is best established in adipocytes where it inhibits lipolysis via the G_i_-protein signaling cascade [46]. In neurons, G_i_ signaling is characteristically associated with the inhibition of action potential activity and transmitter release. Lac has very low potency on HCA1. In the original publication, EC50 for Lac for HCA from different species are listed between 3.7–6.9 mM ([47], Table 1). This is not surprising because, in the periphery, Lac levels in plasma are typically at several millimoles and increase prominently during exercise. Hence, HCA1 sensitivity matches physiological Lac levels (for further discussion see [48]). In the brain, however, according to most sources, average extracellular Lac concentrations do not exceed 1.5–2 mM. While it cannot be excluded that Lac can be more concentrated in microdomains, higher average concentrations of Lac have been reported in pathophysiological situations such as during hypoxia or seizures [48].

The group of J-Y Chatton have published several studies reporting inhibitory effects of Lac on mouse neurons via HCA1 [49,50,51,52]. Using both wild-type and HCA1 knock-out mice, the authors report in their studies the inhibitory effects of Lac and 3-chloro-5-hydroxybenzoic acid (3Cl-HBA), an agonist of HCA1, on neurons in patch-clamp and calcium imaging experiments. Decreases in miniature excitatory postsynaptic potential (EPSC) frequency were also observed. These inhibitory effects of Lac on neurons are explained by the activation of the canonical G_i_-protein signaling pathways by Lac [52]. Moreover, the hippocampal neurons that are modulated by HCA1 were suggested to be excitatory, not inhibitory, since they did not counter-stain for GAD67 [50]. In the latter study, additional experiments were carried out on acute slices from human patients where a reduction in spontaneous EPSC frequency after the application of 3Cl-HBA was found. Overall, the take-home message from this body of work is that neurons in various parts of rodent and human brain express HCA1, and HCA1 activation by Lac inhibits neuronal activity by reducing excitability and via presynaptic mechanisms. While these results are interesting and potentially very significant, Lac-mediated inhibition of excitatory neurons in many areas of the brain is quite difficult to reconcile with many of the experiments where the astrocytic release of Lac is seen to facilitate learning and memory (see Section 2.1 and Section 2.2). The balance in a physiological context between the support of actively firing neurons metabolically or via potentiation of NMDA currents by Lac and their inhibition via HCA1 needs to be considered further. During strenuous physical exercise, plasma concentrations of Lac rise dramatically and Lac can travel from plasma into the brain, thus increasing central Lac levels [17]. Brain Lac concentrations also rise during arousal [53]. If HCA1-mediated inhibition was operational within the physiological range of Lac concentrations, this should result in a shutdown of cortical and hippocampal networks, which clearly does not occur. This suggests that the key questions relate to the relevant concentrations of both Lac and 3Cl-HBA in modulating neuronal activity.

A study by Ordenes and colleagues offers a completely different view of the potential mechanism by which HCA1 can modulate neurons [54]. They studied the arcuate nucleus (ARC) where proopiomelanocortin (POMC) neurons synthesize the anorexigenic neuropeptide α-MSH derived from the POMC transcript. Brain slices used in this study were perfused with ACSF containing 1 mM glucose. This factor (concentration of glucose in the media) can be quite important in many analyses on cultured cells and slices but is not often discussed or considered. Of note, the vast majority of slice studies use solutions with 5 or even 10 mM glucose. This specific study found that ~60% of POMC neurons were activated by 15 mM Lac. Surprisingly, 15 mM D-lactate and 15 mM glucose also activated POMC neurons, but 4-CIN did not prevent the Lac effect, suggesting an extracellular target. The HCA1 agonist 3Cl-HBA (40 µM) also depolarized POMC neurons, and its action could be blocked by pertussis toxin, confirming the involvement of G_i_-protein signaling. Altogether these results point to a role of HCA1, however, the authors could not find HCAR1 transcripts in single-cell transcriptomes of POMC neurons. Instead, using immunohistochemistry, they demonstrate that HCA1 is expressed by local astrocytes. According to the paper, activation of HCA1 on astrocytes leads to a paradoxical increase in astrocytic intracellular Ca^2+^. While Ca^2+^ increase seems an unexpected effect following activation of a G_i_-coupled receptor, it has been reported for astrocytes in other studies [55,56,57]. The authors speculate that this could lead to the release of excitatory gliotransmitters, possibly glutamate, and by this mechanism, POMC neurons may be stimulated by Lac and 3Cl-HBA. With respect to the coupling of HCA1, the current situation is not entirely clear and it is possible that some of the effects are mediated by the βγ complex or another type of Gα subunits, which is not uncommon for G-protein coupled receptors. In addition, the authors argue that if Lac is taken up by POMC neurons, it would lead to increased ATP production, closure of ATP-sensitive K^+^ channels (KATP), and depolarization, although the paper does not appear to contain direct evidence in support of this suggestion [54].

Finally, we consider studies from our own group which also indicate that the brain operates with Lac-sensitive GPCR, but ones distinct from HCA1. We reviewed some of the relevant studies in [48] and now only briefly summarize the current state of play. In 2014, our group demonstrated a link between astrocytes local to a specific subset of noradrenergic neurons in the *Locus coeruleus* (LC; [58]). We concluded that astrocyte-derived Lac stimulates the release of noradrenaline from LC neurons and activates these neurons via a cAMP-dependent signaling pathway. Multiple experiments in that study indicated that Lac does not need to enter LC neurons and that the most logical explanation for these effects was the existence of another, yet uncharacterized, GPCR-mediated signaling pathway which can be recruited by Lac. LC neurons are specialized and different from glutamatergic neurons studied in most other papers. They are rather unique in their morphology and physiology and project all across the central nervous system. Activation of LC is associated with central arousal and active brain states [59,60,61]. Hence, the excitatory effect of Lac on these cells could possibly provide a link between overall brain activity and attention or positive motivation responses and reflect a mechanism by which LC activation by salient stimuli may be amplified to orchestrate generalized cortical desynchronization. Interestingly, we later observed that Lac can also activate another group of noradrenergic neurons in the rostral ventro-lateral medulla that is responsible for activating the sympathetic nervous system, consistent with an autonomic arousal response [62]. We postulated that this effect is mediated by a yet unknown GPCR which has been termed Lac receptor x (or “LLRx”) and is expected to operate via a cAMP-mediated mechanism. Over the years, we have made multiple attempts to identify LLRx and learn more about it but with limited success. For instance, we characterized in a later study its activation by compounds derived from Lac [48].

Apart from HCA1, which is a G_i_-protein coupled receptor and inhibits cAMP production, there are at least two GPCRs sensitive to Lac and known to couple to G_s_-proteins, thus being able to raise cAMP. These are the olfactory receptor OR51E2, which is expressed in several other tissues outside of olfactory epithelium, such as the prostate. We could not confirm the expression of OR51E2 in LC neurons and its pharmacological characteristics do not match what we know about LLRx [48]. By serendipity, we found that the proton receptor GPR4, previously known as GPR19 [63], can be modulated by Lac [64]. GPR4 can probably couple to various G-proteins, but its main signaling partner is G_s_ and, when stimulated, GPR4 leads to profound increases in cAMP [63,64]. GPR4 is expressed by endothelium around the body, including the brain and also by some subsets of neurons, but not the LC neurons. We found that Lac negatively modulates GPR4 and reduces proton-induced cAMP responses [64]. Hence, the characteristics of this GPCR are not consistent with the elusive LLRx but, nevertheless, modulation of GPR4 by Lac could be important for some aspects of brain function.

We undertook a screening effort, analyzed a range of orphan GPCRs that are expressed by LC neurons and found one receptor that could be a viable candidate for further experimentation [48]. In a luminescence assay, the application of Lac within the range of concentrations which we consider physiological (less than 5 mM) to HEK cells expressing GPR137 resulted in highly significant increases in cAMP. 5 mM of Lac elevated luminescence to ~175% relative to control. Moreover, 0.4 mM D-lactate antagonized the effect of 2 mM Lac, consistent with our previously reported observations [58]. In terms of its sequence and splicing pattern, GPR137 is not a typical GPCR. While the typical number of 7 transmembrane regions has been predicted for its amino acid sequence (www.proteinatlas.org (accessed on 1 December 2022), it shares little homology with other GPCRs and its coupling to G-proteins has not been confirmed (IUPHAR guidetopharmacology.org). Nevertheless, its expression is verified by multiple databases, and it has been preferentially localized to the lysosomal compartment (Ensemble, Genecards, NCBI). The potential physiological roles of GPR137 are still little understood. According to in situ hybridization data in the Allen Brain Atlas, there is a widespread expression of the GPR137 transcript in mouse brain (http://mouse.brain-map.org/experiment/show/75651149 (accessed on 1 December 2022). While apparently present in human and mouse, in rat it may not even exist as a full-length protein (https://www.guidetopharmacology.org/ (accessed on 1 December 2022). We believe that the effect of Lac via this receptor should be investigated further.

Similarly, we also found that in cells transfected to express GPR180, Lac could reduce cAMP [48]. While recently it has been shown that GPR180 is not a GPCR but, instead, a component of the TGFβ signaling complex, it may still be an interesting candidate to mediate some Lac effects in the brain [65].

An interesting and entirely unexpected avenue might have been opened by a recent discovery of biological activity of the Lac metabolite N-lactoyl-phenylalanine (Lac-Phe). This product of conjugation of Lac with phenylalanine is formed in the periphery when Lac levels are increased by exercise in mice, humans and racehorses, and can suppress food consumption [66]. This effect strongly suggests a central site of action although the study does not address this possibility. A speculation, that may merit further investigation is that, if Lac in the brain could also be converted into Lac-Phe, some of the central effects previously associated with Lac could actually be mediated by Lac-Phe or a similar metabolite.

To summarize this section, in order to eventually gain a better understanding of the full scale of Lac actions in the brain, there is scope in searching for additional receptors which can be either specifically activated by Lac or modulated by it in a biologically relevant manner.

For the sake of completion, we draw the reader’s attention to yet another possible mode of Lac action, i.e., via lactylation of histone lysines [67,68]. Via this route Lac can potentially induce long lasting epigenetic modifications of gene expression. The relevance of this process for brain physiology is still to be discovered.

## 4. How Do Activated Neurons Engage Local Astrocytes to Release Lac?

As of today, there is no generally accepted theory which explains how astrocytes “know” that the neighboring neurons are active and require metabolic support, or modulation via Lac release is indicated. Moreover, it may be that such a general mechanism does not exist or there is more than one acting together, as it is so often in nature. So, what are the main listed coupling mechanisms between neurons and astrocytes?

The group of L.F. Barros focused on this topic for several years and explains such coupling by the action of elevated extracellular K^+^, NO, glutamate and possibly NH4^+^ [69,70]. Elevation of extracellular K^+^ is a result of neuronal activity and the subsequent opening of voltage-gated K^+^ channels required to restore and maintain the neuronal resting membrane potential. An increase in extracellular K^+^ concentration has a depolarizing effect on the membrane potential of astrocytes, and this could be the signal to activate glycolysis, resulting in Lac output. One concept proposes Na^+^ as a link between extracellular signals, astrocytic energy metabolism and Lac production [71]. Large quantities of glutamate released from excitatory neurons are taken up by astrocytes in a Na^+^ gradient-dependent manner, raising intra-astrocytic Na^+^ that then needs to be extruded by the Na^+^/K^+^-ATPase. The resulting drop in ATP/ADP ratio is proposed as one of the triggers of glucose uptake and anaerobic glycolysis. Hence, according to this view, Na^+^ is the “energy currency” and a “mediator of metabolic signals in the context of neuron-glia interaction” [71], see also [72]. Depolarization of astrocytes could also cause Lac release via opening of the channel described by [9].

An earlier study by M. Nedergaard’s group emphasizes the role of Ca^2+^ as a link between neuronal activation and astrocytic signaling [73]. It was reported that, when neurons are actively discharging, the opening of voltage-gated Ca^2+^ channels results in rapid Ca^2+^ influx into the neurons and thus a drop in its local extracellular concentration. This opens astrocytic connexin Cx43 channels which may release ATP. While that paper does not touch upon the Lac shuttle concept, the release of ATP by an astrocyte can be postulated to feedback to the astrocyte via an autocrine loop, since astrocytes express numerous P2Y receptors and are extremely sensitive to ATP and vigorously respond to it in vivo and in vitro [74], see review by [75]. ATP-triggered Ca^2+^ waves in astrocytes are a well-known phenomenon [62]. In addition, hemichannels in astrocytic membranes can release Lac directly [8]. Hence, coupling via a drop in extracellular Ca^2+^ with the resultant release of ATP could link neuronal activity, astrocytic metabolism, and the release of gliotransmitters together.

Finally, regarding the communication between noradrenergic neurons and glia, the expression of adrenoceptors on astrocytes is well described and confirmed by RNA sequencing [76]. Astrocytes respond to noradrenaline with distinct patterns of Ca^2+^ and cAMP responses that are implicated in gliotransmitter release in general, and Lac release in particular [77,78]. The latter may establish a reciprocal positive feedback mechanism for central noradrenergic transmission (see [58]).

To summarize, currently, we do not have a unifying concept which could explain how the activity of neuronal networks engages astrocytes to release Lac. Development of such is an important goal that requires further evidence linking various mechanisms that have been established to date.

## 5. Future Perspectives

What are the most obvious controversies surrounding the role of astrocyte-generated Lac, where should we move next? The analysis presented here shows that the experimental conditions, concentrations of Lac which are seen as acceptable, tools and interpretation of the results are so different between individual groups that in many cases it is hard to compare them or come to any consensus. We believe that the following questions require a concerted effort of various laboratories, possibly via directly coordinated efforts and sharing of the same tools between them.

Under which conditions are the effects of Lac at concentrations exceeding 3–5 mM physiologically relevant, especially if the impacts of intracellular acidification were not monitored? In that context, when are the changes in the NAD^+^/NADH ratio physiologically relevant?

What is the role of the HCA1 receptor in the brain—is Lac actually inhibitory to many neurons in the cortex and hippocampus and how could this tie together with the proposed role of Lac in the process of memory formation?

What are the local dynamics of Lac in the extracellular space? Can local Lac concentrations significantly exceed the “average” extracellular concentrations reported in the literature? This would require measurements with a new type of genetically encoded biosensor.

Are there any other receptors that are responsive to Lac in the brain, with sensitivity better suited to the reported Lac physiological concentrations (<2 mM)? By what mechanism does Lac excite LC and RVLM catecholaminergic neurons?

What is the trigger for the production of Lac by astrocytes in response to the activation of the neuronal networks? Are there several mechanisms, are they brain area-specific?

This list may be continued. We hope that this review will stimulate and facilitate further collaborative efforts to resolve some of the long-standing mysteries surrounding the roles of Lac in the brain.

## Data Availability

Not applicable.
